# Optimal Cluster Head Positioning Algorithm for Wireless Sensor Networks

**DOI:** 10.3390/s20133719

**Published:** 2020-07-03

**Authors:** Ala’ Khalifeh, Husam Abid, Khalid A. Darabkh

**Affiliations:** 1 Faculty of Electrical Engineering and Information Technology, German Jordanian University, Amman 35247, Jordan; h.abed266@gmail.com; 2 Computer Engineering Department, The University of Jordan, Amman 11942, Jordan; k.darabkeh@ju.edu.jo

**Keywords:** WSNs, mobile sink node, LEACH, path loss

## Abstract

Wireless sensor networks (WSNs) are increasingly gaining popularity, especially with the advent of many artificial intelligence (AI) driven applications and expert systems. Such applications require specific relevant sensors’ data to be stored, processed, analyzed, and input to the expert systems. Obviously, sensor nodes (SNs) have limited energy and computation capabilities and are normally deployed remotely over an area of interest (AoI). Therefore, proposing efficient protocols for sensing and sending data is paramount to WSNs operation. Nodes’ clustering is a widely used technique in WSNs, where the sensor nodes are grouped into clusters. Each cluster has a cluster head (CH) that is used to gather captured data of sensor nodes and forward it to a remote sink node for further processing and decision-making. In this paper, an optimization algorithm for adjusting the CH location with respect to the nodes within the cluster is proposed. This algorithm aims at finding the optimal CH location that minimizes the total sum of the nodes’ path-loss incurred within the intra-cluster communication links between the sensor nodes and the CH. Once the optimal CH is identified, the CH moves to the optimal location. This suggestion of CH re-positioning is frequently repeated for new geometric position. Excitingly, the algorithm is extended to consider the inter-cluster communication between CH nodes belonging to different clusters and distributed over a spiral trajectory. These CH nodes form a multi-hop communication link that convey the captured data of the clusters’ nodes to the sink destination node. The performance of the proposed CH positioning algorithm for the single and multi-clusters has been evaluated and compared with other related studies. The results showed the effectiveness of the proposed CH positioning algorithm.

## 1. Introduction

**Figure 1:**
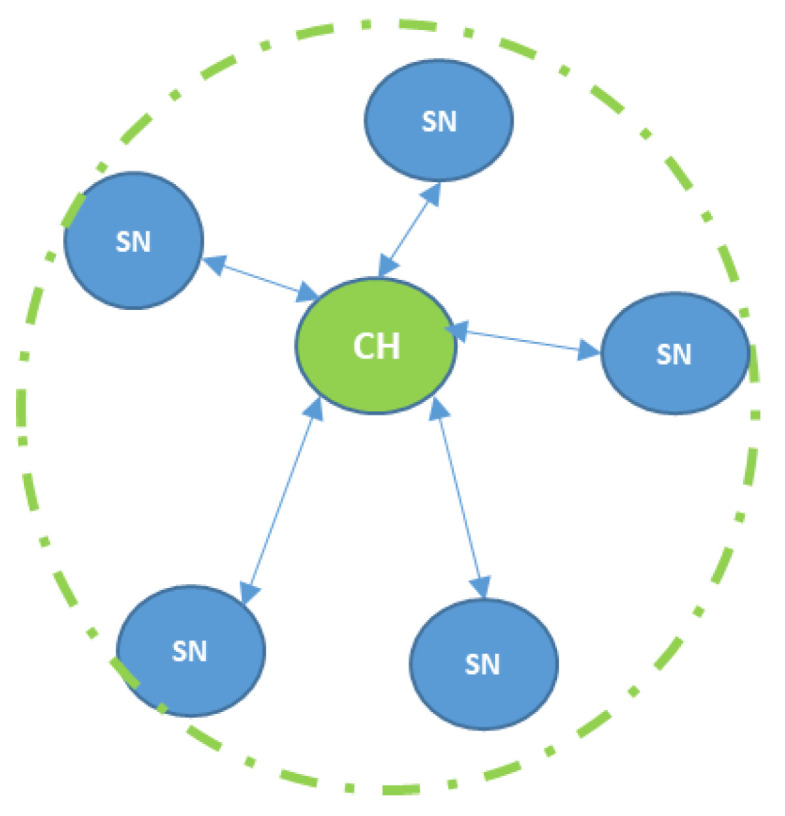
A typical wireless sensor network (WSN).

Wireless sensor networks (WSNs) are types of networks made of a large number of lightweight, small sized sensor nodes (SNs) that are used for sensing and monitoring purposes. The SNs are distributed within a geographical area known as the area of interest (AoI). Further, they cooperate with each other to carry their measured data through the network to the main node, which is called the sink node or base station (BS) [1]. A typical WSN is illustrated in Figure 1, where a group of sensor nodes is collecting data and sending it to a central node named as a cluster head (CH). The CH normally receives and manages the gathered sensed data from the sensor nodes. Further, the CH node normally performs some operations on the collected data according to the application requirements, such as correlation, event trending, status querying and data mining. Afterwards, the CH sends the processed data either directly to the BS or through other CH nodes in a multi-hop manner [2].

**Figure 2:**
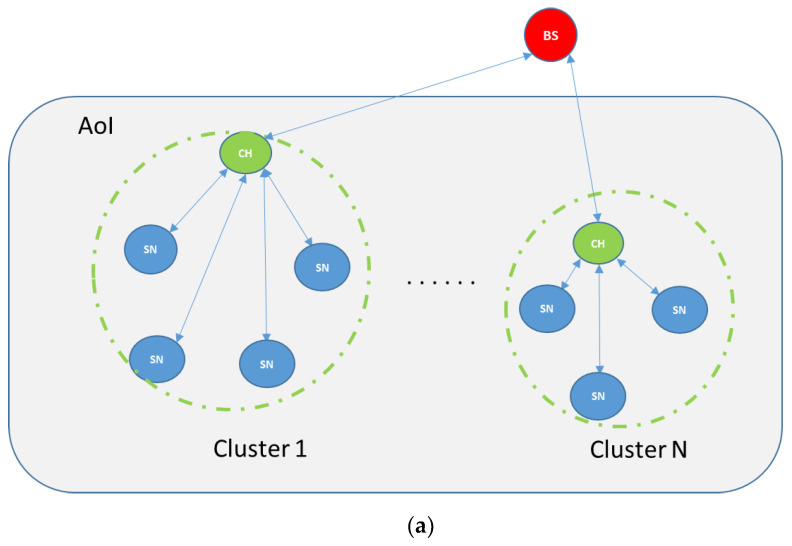

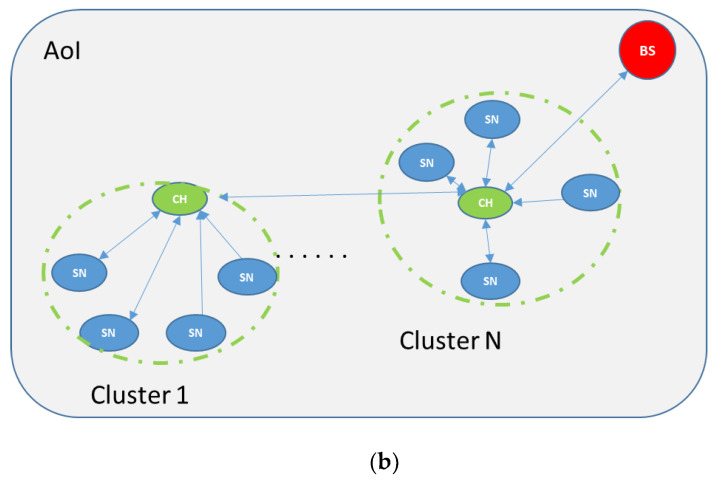
Multi-cluster WSN depicting cluster head (CH) to base station communication through a (**a**) direct or (**b**) an indirect communication via other CH(s) within the Area of Interest (AoI).

In most cases, SNs are powered by batteries; therefore, the lifetime of each one is very restricted and based on its limited energy source. Consequently, the energy consumption is one of the most challenging issues in WSN [1,2]. To address this challenge, several techniques, protocols and algorithms focusing on how to optimize energy consumption, reduce transmission interference and enhance the nodes lifetime have been proposed. To that end, clustering is one of the most efficient techniques widely used in network management for connectivity, coverage and lifetime optimization; whereby, a group of sensor nodes forms a cluster where nodes communicate with each other and send their sensed data to a pivotal node called CH. There are different types of clustering: static or dynamic, single or multi-hop, and homogeneous or heterogeneous. The nodes in the cluster are classified into a cluster master node known as CH, and other cluster sensor nodes known as SNs. Each cluster contains at least one CH, and it is responsible of gathering data from all the clusters’ SNs and sending this data to the sink node or the BS, as illustrated in Figure 2 [3].

The lifetime of running a wireless SN depends mainly on its battery, which is directly related to its power consumption. Obviously, the distance between the SN and the BS or CH is one of many factors that has a direct effect on the sensor power consumption. The limited sensors’ energy in WSNs is one of the main challenges that effects the lifetime of the WSNs. Therefore, once the sensor battery is consumed, the sensor is considered dead. However, not all sensors in WSNs consume their energy in the same level nor die at the same time. Once the first sensor becomes dead, the whole network or cluster will be affected and becomes unbalanced. In this case, not only the data collection process of the monitored area by the dead sensors will be affected, but also the communication paths and data transfer among the cluster or WSN to the CH or BS will be impacted as well. More to the point, another challenge that affects WSN lifetime is the distribution of sensor nodes among the network or cluster, since in most cases, the SNs are deployed randomly in the AoI and are supplied with the same initial energy. However, in hierarchical routing based on clustering, it is the responsibility of CHs to send the information to the sink node, and therefore the CH is supplied with energy greater than other SNs [4].

This paper continues our previous work [5,6,7,8,9,10,11,12] that aims at designing and implementing an energy efficient WSN capable of performing sensing and monitoring functionalities within an AoI. The main contribution of this paper is to propose an algorithm that aims at finding the best CH location, with respect to the other nodes within the same cluster, such that the communication path-loss of all SNs is reduced, and thus the sensor nodes’ lifetime is increased. The proposed algorithm dynamically adjusts the CH location by leveraging CH nodes with mobility functionality to enable them to move to the optimal locations. Introducing mobility to sensor nodes is an emerging research topic, which can be achieved practically, utilizing an unmanned ground vehicle (UGV) or a mobile robot. Further, the proposed algorithm is applied to adjust the locations of multi-clusters CHs located on a spiral trajectory, such that the multi-hop link to the BS is maintained. The proposed algorithm has low complexity and can be implemented practically. The developed algorithm is analyzed and validated through simulation, which outperformed the related work proposed in the literature. The rest of the paper is organized as follows: Section 2 summarizes the most related work. Section 3 illustrates the proposed CH positioning algorithm. The simulation results and analysis are presented in Section 4. Finally, the paper is concluded, and future work is discussed in Section 5.

## 2. Literature Review

In order to enhance the energy consumption levels in WSNs, several techniques, methods and protocols were proposed since the last decade to face the growing need of WSNs in many life applications. Nodes’ deployment, clustering, communication strategies, network topologies and nodes’ mobility are used as key research factors to highlight the energy consumption problem, and thus propose potential solutions. In this section, a literature review of different approaches including nodes’ deployment, clustering, and sink mobility protocols is presented. Moreover, how these approaches can improve the network lifetime in terms of energy conservation, lifetime optimization, and transmission delay is also presented.

### 2.1. Clustering in WSNs

Clustering in WSNs is performed by dividing the sensor nodes into logical groups for load balancing, scalability, energy saving, simplifying management and communication within the SNs and between different clusters. In a clustered WSN, sensor nodes are partitioned into a certain number of clusters, each of which consists of a CH or super node and non-cluster head members or SNs. The role of CH is to collect sensed data from all the cluster members and forward it to other CHs or directly to the BS. However, non-CHs are responsible for sensing the environment and transmitting information to the corresponding CH [13].

There are many different criterions, which can be used for clustering purposes: static or dynamic, single or multi-hop, and homogeneous or heterogeneous. The Low Energy Adaptive Clustering Hierarchy (LEACH) [14] is one of most popular and common clustering algorithms that utilizes dynamic random rotation for the CHs in each round, in order to distribute energy consumption over the network and enhance energy usage during network operation. Using this type of clustering increases network overhead in the setup phase when choosing the CH in each round. One of its concerns is that every node may be a CH regardless to its position in the cluster, even if it is far from the BS, it transmits directly to the BS without using multi-hops. LEACH achieves a large reduction in energy dissipation compared with direct and conventional routing protocols. In addition, it minimizes the number of collisions by using Time Division Multiple Access (TDMA) slots for communication [14]. In [15] the authors proposed a protocol used to maximize the network lifetime, named as the Energy Efficient Data Collection (EEDC) protocol, which is designed for single hop WSNs. EEDC extends the network lifetime using dynamic number of clusters and CHs. In the network set-up phase, the sink node sends a hello message for all nodes within its range, and each node calculates the distance to the BS by measuring the received signal strength, and then selects the proper power amount for communication. In the cluster formation phase, a cluster head node is chosen via the proposed weighted function that ensures energy balancing over the network. Subsequently, the CHs send broadcasting messages to all nodes in their range and the SNs choose which CH to join. Obviously, the authors of [15] focused on the cluster set-up phase to enhance the network lifetime and reduce the total overhead. More to the point, the authors in [16] designed a protocol called Hybrid, Energy-Efficient, and Distributed Clustering (HEED) protocol that handles the coverage and connectivity issues in WSNs clustering, which is a proactive clustering protocol. In this protocol, CHs are selected based on their residual energy, where the node with the highest residual energy is selected as the CH. HEED achieves a connected multi-hop inter-cluster network that maximizes the network lifetime for a determined density model and a specified relation between cluster range and transmission range hold. In [17], Nies et al. provided an overview on computational algorithms for genes clustering with correlated biological functions. Different clustering categories have been summarized, where the pros and cons of each category have been discussed. Further, the authors highlighted the swarm intelligence technique that can be used in clustering, where they showed its effectiveness when compared with the other techniques.

The authors in [18] proposed a Distributed Energy-Efficient Clustering (DEEC) algorithm, where the CH selection is based on the ratio of the residual to average energy of the network. By varying the epoch period, the WSN obtains a well-balanced scheme of CH selection for different types of nodes. The node whose energy level, initial or residual energy is higher gets more chance to become a CH than the node with low energy level. The authors in [19] proposed a novel protocol named Energy Efficient Clustering Protocol to Enhance Performance of Heterogeneous WSN (EECPEP-HWSN) to select the best possible node to be the CH, based on real time information, and to enhance the stability period and lifetime by reducing the energy consumption in the form of reducing internal overhead and cost of processing energy. In the proposed protocol, the SNs are randomly deployed. The issue the authors highlighted here is pertained to load balancing and minimizing energy consumption of the network by dividing the network initially in a subsections (zones) based on the number of SNs. All the nodes have to work for the role of CH but only one at particular cluster round. In the proposed network model, each SN gets an opportunity to work as the CH or intermediate CH rather than the role of cluster member. The sensor or member node plays its role of transferring collected data to respective zone CH, which is located in its coverage range.

### 2.2. Nodes’ Deployment Protocols in WSNs

Nodes’ deployment methods can be classified into static or dynamic. In static deployment, the sensor nodes are deployed using a drone or airplane to a fixed location over the AoI. Data routing is done over pre-determined paths according to the utilized routing protocol. However, in dynamic deployment, the nodes have the capability to change their locations over the monitoring area to enhance the network connectivity and energy consumption. Hence, node deployment is important for building the WSN topology and route the data over the network [20].

In static nodes’ deployment, the locations of the nodes are chosen at the design phase to ensure appropriate coverage of the AoI and suitable connectivity between the nodes. In this approach, the nodes’ locations do not change over time. In static deployment, there are two types of nodes’ deployment: deterministic and random. In WSNs that involve CHs, deterministic deployment is used because the CHs are expensive, equipped with more energy and their operations are significantly affected by their positions [20]. In addition, the use of random deployment is mainly used in militarily and specific applications that cannot change the nodes or move them in the AoI freely. For instance, WSNs deployed in dangerous environments like forest surveillance, earthquake observation, and battlefield where the deterministic deployment of sensors is very risky and/or infeasible [21]. The authors in [21] studied the static nodes’ deployment over a spiral trajectory that is designed for optimal deployment setting in order to achieve full area coverage, energy balancing, and enhanced network lifetime. One of the most challenging problems solved by the spiral trajectory deployment is the excessive energy consumption by the nodes existing near the sink, because they are highly used in most of the routes to the sink. The death of these nodes in an area would cause network holes. The spiral avoids the network holes that disconnect other nodes from the sink in the network. In addition, they address the energy balance and the network lifetime jointly. However, one of the drawbacks of these methods are that they have large overhead and require the CH nodes to have the capabilities to move to the right positions.

The dynamic deployment is different form static deployment as it allows the nodes to change their positions while the network is working to enhance the connectivity, coverage, and lifetime performance. In addition, when some nodes die and stop working because they consumed their energy, other nodes move to cover the dead areas in order to improve the network coverage. As aforementioned, nodes can be deployed randomly or in pre-defined locations. Nodes are deployed randomly because there is no prior knowledge of their required positions at the beginning. In this case, the topology would change to enhance the lifetime, coverage, and connectivity of the network [21]. The authors in [14] proposed a topology scheme to reduce and balance the energy consumption over the network by designing an optimal clustering protocol for self-organizing WSNs. The proposed protocol consists of two phases, clustering, and data transmission. First, the protocol decides the optimal number of CHs, interestingly; each node has the same prosperity to be a CH depending on its residual energy, such that the energy consumption is balanced over the network. Since the distance between CH and BS is usually long, the protocol uses a multi-path model. Ultimately, when the CH is chosen, then a tree is built with child nodes and CH acting as its root. Once data is collected, the CH transmits this data to the sink node. Hence, the proposed scheme reduces the energy consumption and enhances the network lifetime.

In this paper, the proposed algorithm uses a hybrid deployment method for SNs, where the CHs nodes and the BS are deployed in pre-determined locations over spiral trajectory to take the benefits of convergence and connectivity. Additionally, the SNs are randomly distributed around the CH nodes. However, to optimize the CH nodes locations relative to the SNs, the CH nodes are assumed to be mobile nodes that can adjust their locations to minimize the SNs energy consumptions and reduce the CHs to SNs’ path-loss.

### 2.3. Sink Mobility in Wireless Sensor Networks

To enhance the energy utilization level of wireless SNs, the integration of mobile SNs is carried out in WSN. In many works, such as in [22,23,24], the authors introduced the implementation of mobile SNs in WSNs to enhance the energy consumption. There are three main models involved in implementing sink mobility to WSNs to improve the energy utilization: sink node movement, data packets routing, and data gathering. Additionally, in our proposed algorithm, we assume the BS has a fixed location, and the CHs have the capability to change their locations. In [23], the authors proposed a data-gathering model cooperatively with a random walk strategy to save energy and collect sensor data in sparse sensor networks. In this model, the SNs can reduce their transmission ranges to save energy. The authors in [24] proposed a BS location computation method using integer liner programming (ILP) to enhance the network lifetime and data throughput. Interestingly, this is done by considering the BS located not at the network center, but at the boundary of the network area. In [25], the authors proposed a novel optimized clustering framework to investigate the effects of sink mobility on the network lifetime. Furthermore, they proposed two routing protocols based on the spiral mobility pattern: Spiral Mobility Based on Optimized Clustering (SMOC) for optimal data extraction, and Multiple Sink based on SMOC (M-SMOC) for large-scale WSNs. In SMOC, a single mobile sink moves in a predefined spiral track over the sensing field to collect data from sensor nodes and CHs, while in M-SMOC, four mobile sinks move in the spiral tracks over the sensing field to cover the whole network area and avoid the delay in long-range communication with CHs. In our proposed algorithm, the CHs are distributed in a spiral trajectory within the AoI, where the cluster nodes (i.e., SNs) are deployed randomly and clustered around the CH nodes. This makes the nodes’ deployment and distribution more optimal in covering the AoI [26], while in SMOC, the nodes and CHs are randomly distributed in the AoI, which may result in scenarios with lower sensing coverage and connectivity issues [25]. Further, the CHs adjust their locations within their cluster area, which results in a very limited mobility; thus, making the proposed algorithm more realistic and applicable compared with other related work that assumed a large-scale mobility for the CH, which may not be feasible or practical from implementation point of view.

To counter the problem of data gathering in sparse sensor networks in many applications such as monitoring physical environments, weather conditions in national parks, city traffic monitoring etc. [27].

The authors in [28] proposed a Mobile Sink Based Routing Protocol (MSBRP) as a cluster-based routing protocol. MSBRP clusters the network and selects the cluster head based on the residual energy information on each node. The dynamic mobile sink architecture in MSBRP avoids energy concentration on a small number of SNs and prolongs the network lifetime. Based on the residual energy of cluster heads, the sink will always move to be close to the cluster head with higher residual energy than others. In [29], the authors proposed a dynamic routing protocol that makes routing decisions locally when selecting a neighboring sensor node depending on two factors: the average energy and distance in transmission. They first divide the whole sensor area into multiple clusters. In each cluster, a CH is selected according to the residual energy and the distance between the source node and the CH. Source nodes communicate with the CH using single or multi-hop communication in accordance with the optimal energy consumption. Then, the mobile sink moves along a predefined trajectory for data gathering.

One important issue in implementing mobile sink nodes in wireless sensor networks is how the sink node will gather the data from static sensor nodes while it is moving. As the location of the sink is changing, sensor nodes are enabled to send the data packages to the sink when the sink is nearby. Therefore, traditional data gathering, and routing schemes are not suitable in this case. The authors in [23] proposed the idea of using multiple mobile elements instead of one or a static sink, where multiple mobile elements are used to collect data while moving on predetermined paths. Mobile elements will stay at certain points that are fixed to gather data from sensor nodes. This algorithm splits the network into two important areas to gather data, the first area is concentric sphere of deployed region with radius (*r*), and the other area is divided into eight sub-areas. The mobile elements move along the diameter of the sphere and other two sinks which are present, move along the arc lines to gather the packet data of SNs.

The authors in [30] proposed a data gathering hierarchy scheme, where two types of mobile SNs were utilized. The first type is called a mobile collector (MC) node, which is used to collect data from SNs, where the second type of nodes is the mobile relay (MR) node that collects the data from the MC nodes and transfers it to the sink node. It can be observed from the conducted literature review that most of the published work focus on proposing algorithms and frameworks related to CHs and BS deployment during the network initialization and setup phase, without dynamically adjusting the CHs locations during the network operation. Another energy efficient data gathering and critical event detection WSN was proposed in [31], where the focus was to send the gathered data and the critical events to the sink node utilizing multi-hop communication link. However, the paper did not address the CHs locations optimization and how path-loss between the CH and the nodes can be reduced.

In this paper, the proposed algorithm works on repositioning the CH nodes frequently during the network operation lifetime, such that, the new CH position results in minimizing the path-loss between the CH and the sensor nodes. This paper extends and further explains our previous paper [5], where only the case of a single cluster is discussed. Further, to mitigate interference between the SNs, a distributed TDMA mechanism is used, where each SN is assigned a time slot in order to avoid collision and congestion between the nodes. The new proposed approach is based on leveraging CH nodes with mobility, such that they can dynamically and frequently change their locations thus improving the nodes energy efficiency within the cluster and the entire network.

## 3. CH Positioning Algorithm

In this section, a WSN Cluster Head Positioning algorithm (CHP) is proposed, which aims at reducing the total path-loss among all communication links between the SNs and the CH within the cluster. Further, it aims at reducing the interference between nodes and the overall energy consumption such that the overall network performance and the network lifetime are improved. Intriguingly, the algorithm is considered in both single and multi-cluster WSNs, while achieving a better lifetime in all nodes in the network. This is done by considering the effect of the sink node’s location in reference to all SNs in the AoI. Because of its advantages over other network topologies, the multi-cluster algorithm is applied on a spiral trajectory network; such advantages can be summarized by the reduction of network interference, enhancement of network coverage and nodes’ deployment criterion and reduction of energy consumption. The performance of this algorithm is validated mathematically and by simulation, using MATLAB, and is compared with the related work in literature.

This work addresses the problem of finding the optimal location of the CHs for enhancing the network energy consumption and network lifetime for single and multi-cluster WSNs. Given a set of sensor nodes {*SN*_1_, *SN*_2_, …, *SN_N_*}, where *N* is the number of sensor nodes, distributed in random locations (*x_i_*, *y_i_*) in an AoI, and the CHs positions are predefined, with the ability to change their locations automatically (mobile nodes). In the proposed algorithm, sensor nodes are heterogeneous. Hence, there are two types of nodes: super nodes with high battery supply deployed with mobility capability, and SNs that have limited capabilities with a limited battery source and fixed random locations. There is also one fixed sink node location located at specific coordinates that does not have any energy limitation, as this node is normally located far from the AoI (e.g., operation monitoring center) and is assumed to be connected to a permanent power source. The SNs are organized into clusters. After the clusters’ formation process, the CHs broadcast request messages for all SNs within the cluster about their locations and IDs. Each CH calculates its optimal new location that gives the minimum summation of path-loss of all the links between SNs and their CHs. The process is performed by exchanging some control messages between CHs and SNs. Notice that the payload for these control messages has a small size, since they carry the nodes’ position coordinates. Therefore, they can be piggybacked with the data messages, which are sent frequently to the CH node; thus, reducing the control message overhead.

The proposed algorithm finds the best location of a CH in a single cluster, and the best formation of a given number of clusters distributed in a spiral topology, the CHs communicate with each other until the data reaches the BS. For each case, the power consumption and the network lifetime are considered as a metrics for evaluation purposes. In this work, the proposed algorithm is applied first into the nodes within the same cluster, and then the algorithm is evaluated and tested under a multi-cluster spiral trajectory network. The following sections explains both the case of single and multi-cluster WSN.

### 3.1. Single Cluster Head Positioning Algorithm

In this work, the Friis free space model [14] is assumed, where a free space path-loss for a certain path is proportional to the square of transmission distance between SN and CH. However, multi-path fading channel model should be used for large distances, where the path-loss is proportional to the fourth power of transmission distance [32].

**Figure 3:**
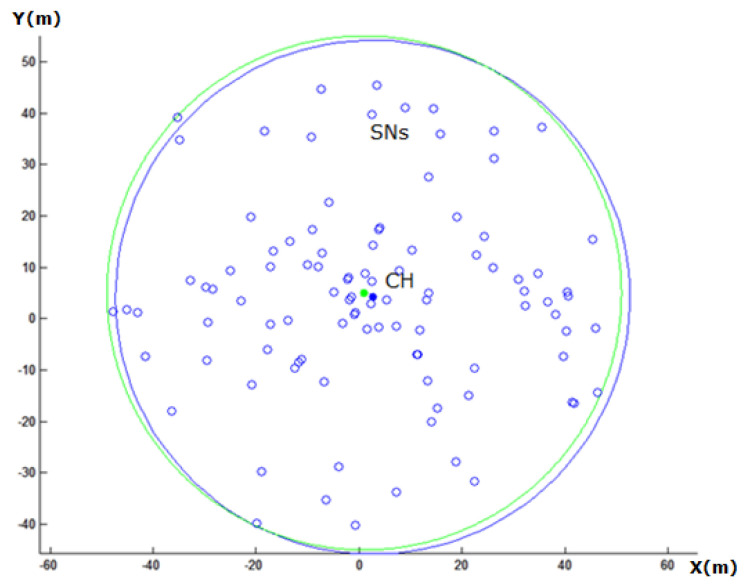
The CH position before and after the adjustment, marked with a dot colored with blue, green, respectively.

The CH is a mobile node and can change its location and adapt its coverage area to keep the connection with any SN that may get out from its coverage range. The blue solid circle in Figure 3 illustrates the current location of CH with 30 m radius as a coverage range, which is similar to the other nodes’ coverage range. While the green one depicts the optimal location of CH, which ensures the minimum path-loss for all nodes and better lifetime for the cluster. The blue, green, big circles illustrate the coverage of the CH before and after the CH movement, respectively.

For a given single cluster with *N* non-CHs nodes, the position of each node is represented as ($x_{i}$,$y_{i}$) where *i* = 1, 2, …, *N*. The objective of the algorithm is to find the optimal location for the CH leading to the minimum overall path-loss and energy consumption of the cluster nodes. From the Friis free space model, the energy consumption of a WSN can be calculated as follows:

(1) $$E_{CH}=lN(E_{elec}+E_{DA})+l\varepsilon_{fs}*d_{to\;BS}{}^{2}$$

(2) $$E=\sum_{k=1}^{N}E_{\left(non-CH\right)k}=lE_{elec}N+l\varepsilon_{fs}*\sum_{k=1}^{N}d_{\left(to\;CH\right)k}{}^{2}$$

where
ETotal energy consumption for all non-CH member nodes (J),*l*No. of bits on each data message,$E_{DA}$Energy consumption for data aggregation (J/bit/signal),$E_{elec}$Energy consumption for processing each bit of data (J/bit),*N*No. of nodes on each cluster,$d_{to\_BS}$Distance between the CH and BS (m),$d_{to\_CH}$Distance between each node and the CH (m),$\varepsilon_{fs}$Coefficient of amplifier energy in free space model (J/bit/$m^{2}$),$E_{CH}$Energy consumption for the CH (J), and$E_{\left(non-CH\right)}$Energy consumption for non-CH member node (J).

Let: $A_{1}=l\;\left(E_{elec}+E_{DA}\right)$

$$A_{2}=l\;E_{elec}\;A_{3}=l\varepsilon_{fs}$$

Substitute $A_{1},\;A_{2},\;A_{3}$ in Equations (1) and (2):

(3) $$E_{CH}=A_{1}N+A_{3}d_{to\_BS}{}^{2}$$

(4) $$E=\sum_{k=1}^{N}E_{\left(non-CH\right)k}=A_{2}N+A_{3}\;\sum_{k=1}^{N}d_{\left(to\_CH\right)k}{}^{2}$$

We assume that the BS location coordinate is (0, 0), and the optimal location of CH is (*x*, *y*). The total energy consumption ($E_{total}$) by all SNs can be found by adding Equations (3) and (4) as shown below:

(5) $$E_{total}=E_{CH}+\sum_{k=1}^{N}E_{\left(non-CH\right)k}$$

(6) $$E_{total}=N\left(A_{1}+A_{2}\right)+A_{3}d_{to\_BS}{}^{2}+A_{3}\;\sum_{k=1}^{N}d_{k\left(to\_CH\right)}{}^{2}$$

where $\sum_{k=1}^{N}d_{k\left(to\_CH\right)}{}^{2}=\sum_{k=1}^{N}[{\left(x_{k}-x\right)}^{2}+{\left(y_{k}-y\right)}^{2}]$

$$\mathrm{and}\;d_{to\_BS}{}^{2}=\left(x^{2}+y^{2}\right)$$

Then we can write $E_{total}$ as:

(7) $$E_{total}=N\left(A_{1}+A_{2}\right)+A_{3}\left(x^{2}+y^{2}\right)+A_{3}\;\sum_{k=1}^{N}[{\left(x_{k}-x\right)}^{2}+{\left(y_{k}-y\right)}^{2}]$$

We can write $E_{total}$ as a form of F(x,y) as follows:

(8) $$F\left(x,y\right)=A_{3}\left(x^{2}+y^{2}\right)+A_{3}\;\sum_{k=1}^{N}[{\left(x_{k}-x\right)}^{2}+{\left(y_{k}-y\right)}^{2}]$$

To find the optimal solution of F(x,y) that will achieve minimum energy consumption, the following equations must be solved:

(9) $$\frac{\partial F}{\partial x}=2xA_{3}-2A_{3}\;\sum_{k=1}^{N}\left(x_{k}-x\right)=0$$

(10) $$\frac{\partial F}{\partial y}=2yA_{3}-2A_{3}\;\sum_{k=1}^{N}\left(y_{k}-y\right)=0$$

From the last two equations, we can easily find the optimal location (*x*, *y*) as follows:

(11) $$x=\frac{\sum_{k=1}^{N}\left(x_{k}\right)}{N-1}$$

(12) $$y=\frac{\sum_{k=1}^{N}\left(y_{k}\right)}{N-1}$$

**Figure 4:**
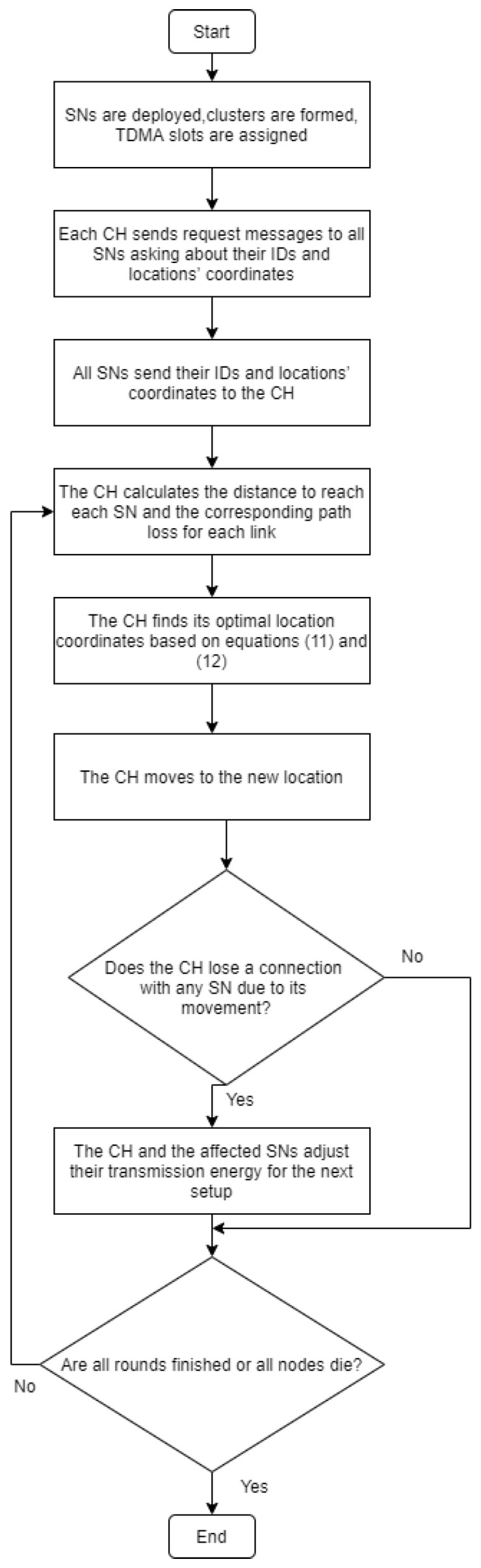
The flow chart of the Cluster Head Positioning algorithm.

After the CH is moved to its optimal location, which is calculated from Equations (11) and (12), some nodes may become unable to communicate with the CH. Therefore, after the CH moves, it discovers which nodes became out of its coverage area; thus, both the CH and the out of range node increase their transmission energy to cover all out of range nodes as illustrated in Figure 4.

As depicted in Figure 4, the CHP algorithm starts after the cluster is formed in the following sequence. The CH first sends a request message to all SNs within its cluster, asking about their locations and IDs. Once the CH receives the reply messages form the SNs, it starts calculating the optimal location based on Equations (11) and (12), then the CH moves to the new location. Next, the CH will check if any SN becomes out of its range because of its movement. If there is any, the CH will extend its coverage radius by a value proportional to the distance moved; thus, increasing its transmission energy to discover these nodes again. At the end, this process will be repeated every iteration if there are dead nodes. The path-loss is defined as the reduction in power density of an electromagnetic wave as it propagates through space. Friis transmission formula [32] defined in Equation (13) is used for calculating the signal path-loss between the feed points of two isotropic antennas in free space:

(13) $$\mathrm{Path}-\mathrm{loss}=20\;\log\left(\frac{4\pi d}{\lambda }\right)$$

where the path-loss is calculated in dB, λ is the wavelength, and *d* is the transmitter–receiver distance in the same units as the wavelength.

### 3.2. Multi Clusters Head Positioning Algorithm

**Figure 5:**
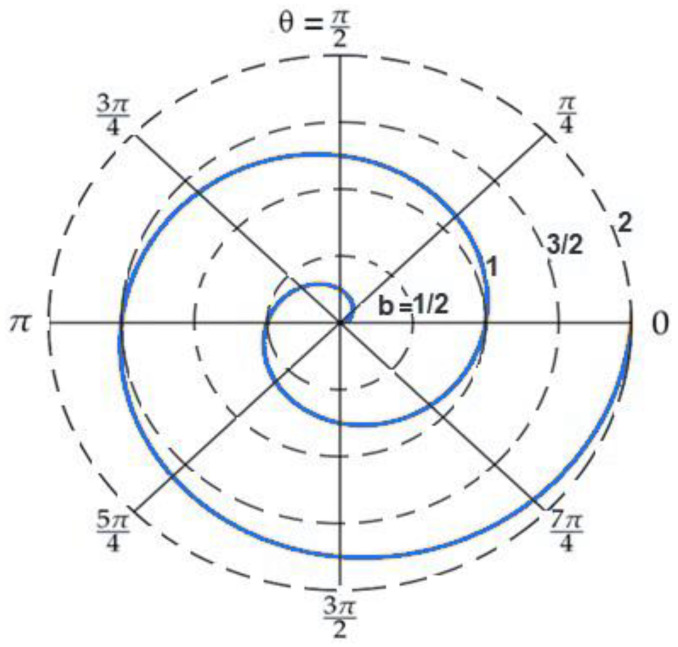
Spiral pattern.

The CHP algorithm proposed for single cluster is applied and evaluated for the multi-cluster case. In which the CHs are distributed on a predefined spiral trajectory, as illustrated in Figure 5, and can transmit to the BS through ad-hoc communication with other CHs. The reason behind adopting the spiral trajectory for the CHs is that it has proven its effectiveness in enhancing the network lifetime [26]. The objective is still to minimize the overall energy consumption. The main idea of the proposed algorithm is to find the optimal CHs’ locations with respect to the clusters’ SNs on the predefined spiral trajectory, with a lower overall energy consumption. However, the optimized CHs location should maintain the inter-cluster communication link over the spiral trajectory path, which is achieved by adjusting the CHs energy transmission ranges appropriately.

In Figure 5, each circle is called a layer. A CH is located on a layer where it sends the gathered data from its SNs to the next CH in the next layer until it reaches the sink in a multi-hop approach. The distance between the sink and the CHs is always known. Every CH should be placed in the range of at least one other CH. The spiral trajectory used is a continuous spiral with polar coordinates (*r*, *θ*) shown in Equation (14), which can be described by real numbers *a* and *b*. Changing the parameters *a* and *b* will turn the spiral and control the distance between successive turnings, respectively [33]. Notice that in order to avoid potential interference between the clusters, especially in the inner spiral cluster nodes (i.e., SNs), different communication channels are used within the clusters, which drastically alleviates the interference problem.

(14) r=a+bθ

**Figure 6:**
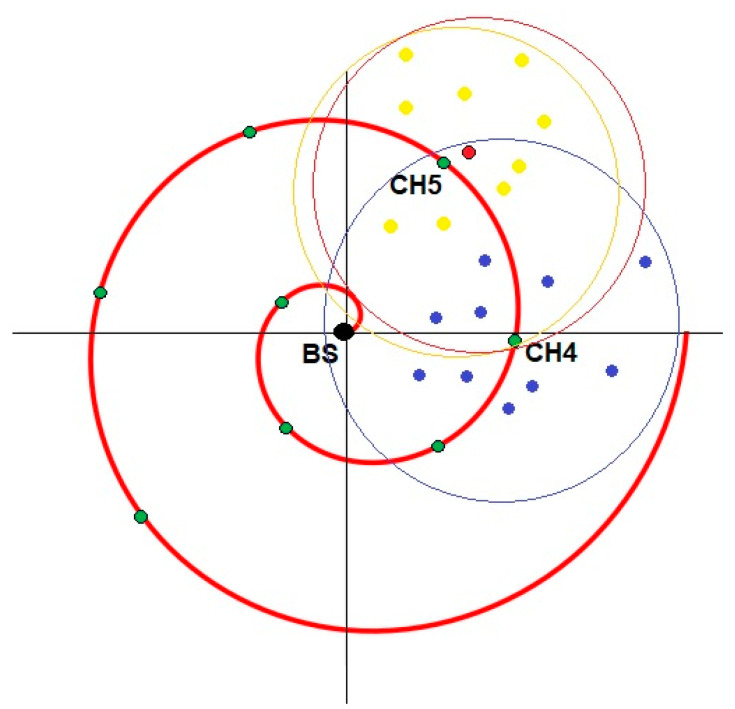
Multi-cluster head positioning algorithm (CHP) example.

The best CHs locations are calculated where each of the nearby CHs is serving as the next-hop node in ad-hoc transmission to reach the BS. The algorithm for finding the optimal position of CH for ad-hoc transmission through other CHs is a simple extension of the algorithm for finding optimal CH location for single cluster as follow:

The multi-CHP applies the same steps as CHP with some additions to ensure the connectivity with next hop CH. For example, in Figure 6, CH5 starts the proposed algorithm to find its optimal location by broadcasting messages to all the SNs asking about their locations. Once it receives replies from all SNs in the cluster, CH5 calculates the optimal location to conserve energy, the small red circle in the example illustrates the optimal location for CH5. Subsequently, CH5 moves to its new optimal location and starts adapting its transmission range to keep the connectivity with all cluster SNs and next hop CH (CH4 in this example). The optimal location is calculated according to Equations (11) and (12). Further, it is important to mention that since the CH nodes are allocated in pre-defined locations identified by the spiral trajectory; each CH is aware of the next hop CH that will relay the traffic ultimately to the sink node in a multi-hop paradigm. Therefore, static routing is used, where each CH identifies the next hop CH by its ID. Further, each CH will send its data to the next CH at the end of each transmission round, i.e., once it receives all the sensors’ data within its cluster. It is noteworthy to mention that despite the fact that the CH node has high energy compared to the other nodes, it is still important to optimize its location to preserve the other nodes from depleting their energy quickly. Notice that the SNs are deployed randomly around the spiral trajectory positioned CHs, where the nodes will connect to the closest CH; thus, forming several clusters. In conclusion, the network topology is a mixture of fixed infrastructure nodes with relaying functionalities between the CH nodes distributed over the spiral trajectory, and randomly deployed nodes around the CH nodes forming several clusters.

## 4. Simulation Results and Analysis

In this section, the performance of the proposed algorithm is evaluated. The simulation results show considerable advantages of the proposed algorithm compared to that of other related work. This section is organized as follows: First, the simulation results of repositioning the CH of a single cluster for different cluster sizes and transmission rounds is illustrated. These experiments aim to show the effect on network performance of moving the CH to the calculated optimal location based on the proposed algorithm. Several metrics are used to analyze and compare the performance of the proposed algorithm such as the total number of alive nodes, total number of dead nodes, and energy consumption. Further, two scenarios are examined and compared, the first one considers the case where the CH is static, and thus cannot optimize its location, while the second scenario shows the case where the CH node can move to the optimal location. Afterwards, the performance of the algorithm is examined under the case of multi-cluster network, where CHs are positioned in a spiral trajectory all the way to the BS.

In order to show the effectiveness of the proposed algorithm, a performance comparison with the LEACH protocol and other related work is performed. LEACH is one of the most widely used protocols in WSN, used in many research as a benchmark to evaluate the performance of WSN operation in terms of network lifetime, and energy consumption. Finally, in the rest of the sub-sections, the performance of the CH positioning algorithm (CHP) and multi-CHP are compared with the dynamic base station-positioning algorithm [34], SMOC [25], DEEC [18], and the EECPEP-HWSN [19] in terms of energy consumption and number of alive nodes after certain number of transmission rounds.

### 4.1. Single Cluster Head Positioning Algorithm

The proposed algorithm was simulated using MATLAB environment and compared with the results of the LEACH protocol and other related algorithms. All the simulation results were an average of 1000 runs. Comparison was performed in the number of alive nodes, energy consumption of the network, and the path-loss for all connections. Table 1 shows the simulation parameters used for the next two scenarios.

#### 4.1.1. First Scenario: Multiple Transmission Iteration Rounds with Varied Number of SNs

**Figure 7:**
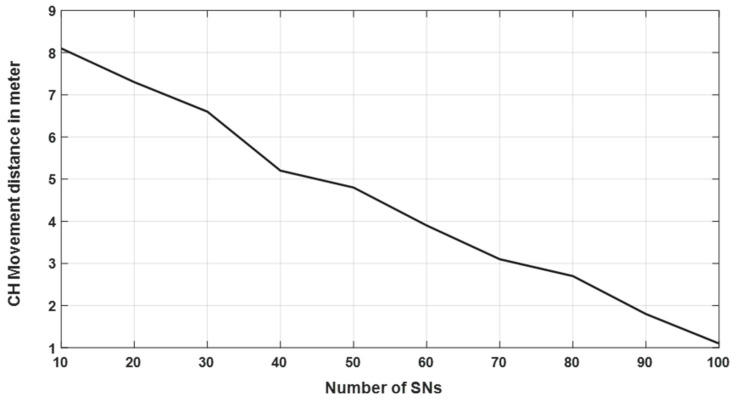
Distance in meters that the CH needs to move to the optimal location.

We assumed a 100 × 100 m^2^ AoI with SNs scattered randomly from 10 to 100 nodes for testing. Simulations were running with the nodes randomly distrusted in the AoI around the CH, which has a predefined location in the AoI. This was considered to have a close correlation with the real-scenario, where the CH (which is a mobile sensor node) is deployed in a pre-defined location, for example, at the center of the AoI where the sensors were planned to be deployed, whereas the other static nodes were randomly distributed over the AoI. As shown in Figure 7, the distance for the CH that needs to move to reach the optimal location is calculated based on Equations (11) and (12).

As illustrated in Figure 7, the distance that the CH node moved is inversely proportional to the number of cluster sensor nodes. That is, when the number of nodes increased, the distance that the CH needs to move to the optimal location decreased. This result was expected because the probability that the CH was close to the optimal location was high due to the large number of sensors in the cluster.

Furthermore, the results obtained in Figure 7 were calculated in the following order. *N* nodes were distributed randomly around the CH node, which was assumed to be deployed close of the center of AoI with an area equals to 100 × 100 m^2^. The optimal location for this CH was calculated according to Equations (11) and (12). After that, the distance that this CH has to move was calculated. The experiment was repeated 1000 times, and the average distance the CH moved was estimated. These experiments were repeated for different node numbers ranging from 10 to 100, as depicted in Figure 7.

**Figure 8:**
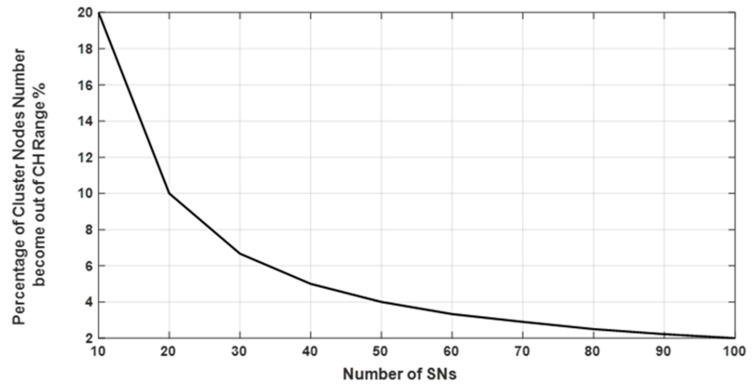
The percentage number of nodes that lost their connections with CH after CH movement.

In Figure 8, the percentage number of SNs, which came out of the CH coverage range is illustrated. Because of CH movement, as shown in Figure 3, some nodes may have lost their connectivity with the CH. For the network with a large number of SNs, the percentage of SNs, which came out of the coverage range of CH, was smaller in comparison with small network.

**Figure 9:**
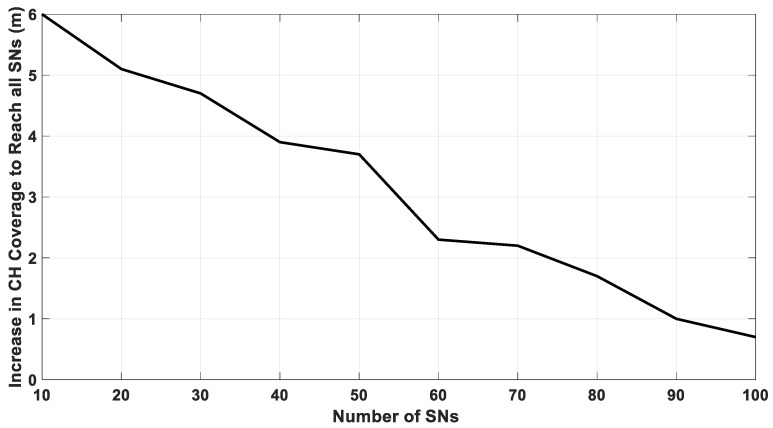
Coverage in meter that the CH needs to adjust to access all sensor nodes.

Figure 9 shows the increase in coverage in meters for the CH and the SNs, which lost their connection with the CH, to keep all cluster nodes connected with the CH. As illustrated in Figure 9, the increase in coverages depends on the cluster size, for large number of nodes the change in the coverage range becomes smaller.

**Figure 10:**
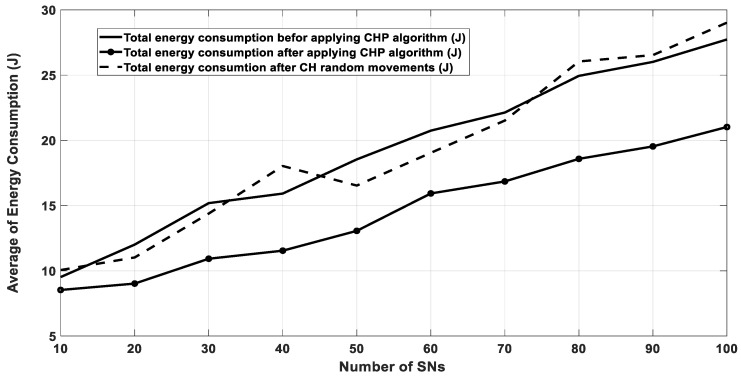
Energy consumption for all the SNs after 1000 rounds.

Further, Figure 10 depicts the energy consumption after 1000 transmission rounds for three possible scenarios. The first one, the CH did not move; the second scenario, the CH moved to a new location randomly; and finally, the CH moved to its optimal location with respect to its SNs utilizing the CHP algorithm. In all the three scenarios, all sensor nodes started to run with an initial energy equals to 0.5 J and CH with 2.5 J, as mentioned in Table 1. The figure shows clearly that the proposed algorithm significantly saves energy due to path-loss optimization.

#### 4.1.2. Second Scenario: Multiple Transmission Iteration Rounds with A Fixed Number of SNs

Similar to the previous scenarios, the AoI was assumed to be 100 × 100 m^2^ with a fixed number of SNs (40 in this case), scattered randomly for testing purposes, simulation rounds were varied from 100 to 2000. Simulations were run using MATLAB, where the CH’s initial position was set around the AoI center, and the static SNs are randomly distrusted in the AoI.

To ensure a fair comparison, the total initial energy for all nodes for CHP was equal to the total initial energy for the SNs in LEACH protocol. The initial energy was equal to 22.5 J, which was distributed as follow:

- each SN in LEACH protocol had an initial energy equals to 0.5625 J,
- each SN in CHP had an initial energy equals to 0.513 J, and
- the CH had an initial energy equals to 2.5 J.

**Figure 11:**
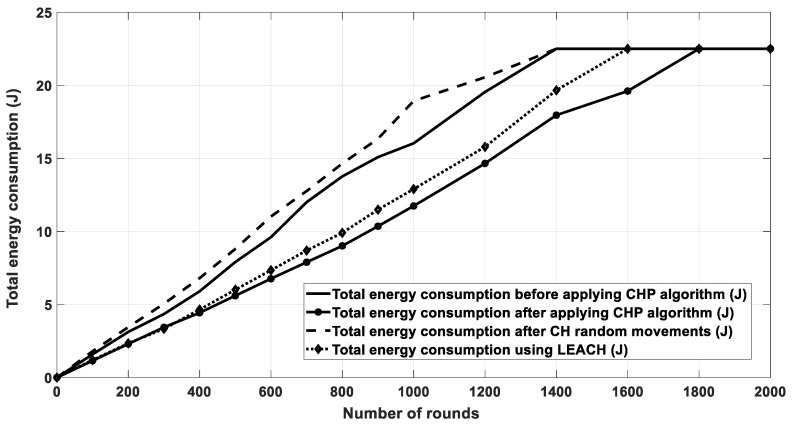
Energy consumption for all the SNs after each round.

**Figure 12:**
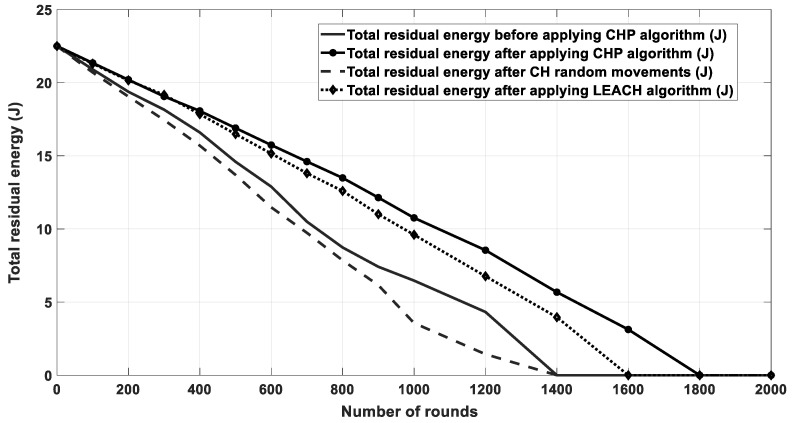
Remaining energy for all the SNs after each round.

Figure 11 shows the results of energy consumption for all nodes in each round in the cases of LEACH protocol. As depicted in the figure, when the number of runs was low (400 and below), the LEACH protocol had similar performance to the proposed algorithm. This happened because at the beginning, all the nodes still had a plenty of energy and only small energy was consumed due to the small number of data transmissions. However, once the number of transmission rounds increased, more data transmission took place and the nodes consumed more energy; thus, the effect of optimizing the CH location and reducing the consumed energy became more observable in the higher rounds. Further, Figure 12 shows the remaining energy for all nodes after each transmission round. For example, the enhancement percentage in energy consumption of CHP is around 4% compared to the LEACH protocol.

**Figure 13:**
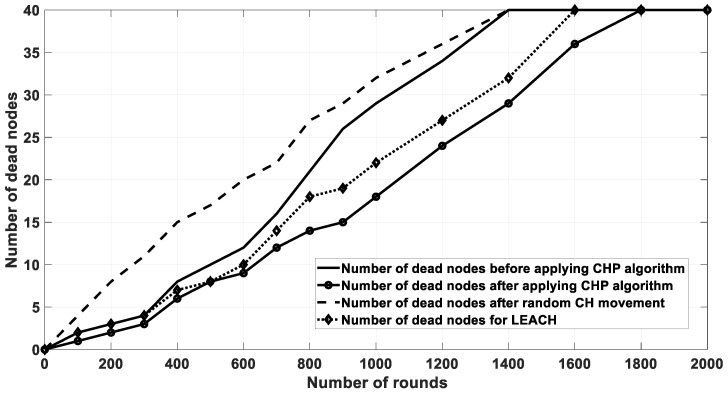
The number of dead nodes after each iteration.

The last comparison for this scenario considered the number of dead nodes after each round. The node becomes dead if it consumes the majority of its transmission power. This affects the network lifetime. In this work, the network lifetime was estimated when all nodes in the network consumed the majority of their energy. Furthermore, based on Figure 13, Table 2 shows the first node to die (FND), half node to die (HND), and last node to die (LND) performance metrics. These were defined as the number of rounds when the first node, half nodes, and all nodes died, respectively. As shown in the table, the FND, HND, and LND were the largest for the proposed algorithm (after applying the proposed CHP movement), which indicates that the proposed algorithm preserved the nodes energy for larger number of rounds (i.e., longer periods) than with that of the other scenarios.

### 4.2. Multi Cluster Heads Positioning Algorithm

**Figure 14:**
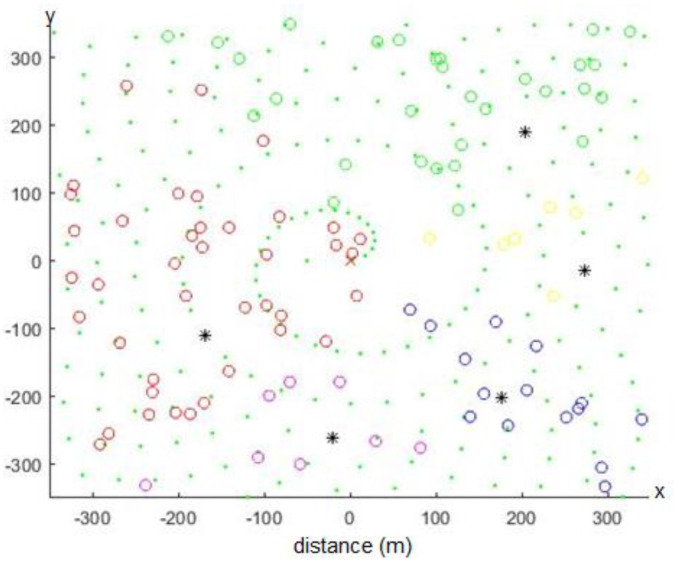
WSN spiral network topology, CHs noted with (*) are deployed on a spiral trajectory.

In this section, the same algorithm for repositioning the CH in a single cluster network was applied for the scenario of multi-clusters, where the CHs were deployed over the spiral trajectory as described before. In this case, the CHs first optimized their locations with respect to their cluster nodes, and then adjusted their transmission range to cover all the SNs and the next hop CH deployed over the spiral trajectory. This is important in order to convey the captured data to the base station. The simulation setup and parameters used were the same as the ones in Table 1 and in the single cluster scenario with the following changes. The total number of nodes was set to 600; the AoI was equal to 300 × 300 m^2^, while the cluster heads were distributed on a trajectory spiral path to cover the entire network as shown in Figure 14. Notice that according to the LEACH protocol [14,35], the number of CHs was equal to 5% of the total number of nodes. For the other nodes’ distribution over a spiral trajectory, we utilized the optimal number of CHs (*kopt*) calculated using Equation (15) [36]:

(15) $$kopt=\sqrt{\frac{N}{2\pi }}\cdot \frac{2}{0.765}$$

In the conducted simulations, the total number of nodes *N* equals to 600, which resulted in the following number of CHs:

- For LEACH, the number of CHs = 5% × 600 = 30.
- For the spiral CHs distribution, the number of CHs equals to 26 according to Equation (15).

The total initial energy for all nodes of the multi-CHP equals to the total initial energy of the SNs in LEACH protocol. The initial energy was set to 350 J, which is distributed as follows:

- each SN in the LEACH protocol has an initial energy equals to 0.583 J,
- each SN in multi-CHP has an initial energy equals to 0.5 J, and
- each CH has an initial energy equals to 2.432 J.

The black stars in Figure 14 are the CHs, which are located over the spiral trajectory, and the colored empty circles are the SNs, which are randomly distributed over the AoI. However, each cluster has a specific color. Further, the green dots represent the spiral trajectory.

**Figure 15:**
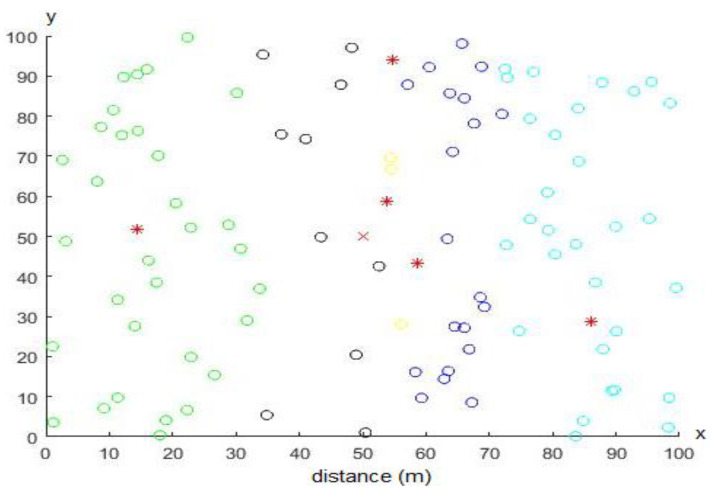
An example of LEACH network topology with an area of interest (AoI) equals to 100 × 100 m^2^.

On the other side and for comparison purposes, the LEACH protocol was simulated with the same parameters used in Table 1. Figure 15 shows an example for the distribution of sensor nodes to be clustered and simulated using the LEACH protocol utilizing an AoI equal to 100 × 100 m^2^. A random topology distribution is used in the LEACH protocol where the CHs are depicted by red stars, while SNs are depicted by empty circles. Every cluster has a specific color.

The next three figures show the results of simulating the multi-CHP algorithm and the LEACH protocol by deploying 600 SNs over 1000 transmission rounds in an AoI equals to 300 × 300 m^2^. The optimal number of CHs (kopt) [36] in both cases was calculated using Equation (15), which was equal to 26 nodes, while it was equal to 30 for LEACH. In the proposed algorithm, the CHs communicated with the sink node through each other in a form of ad-hoc network till the packets arrived at the sink node, while in the LEACH protocol, the CHs communicated directly with the sink node.

**Figure 16:**
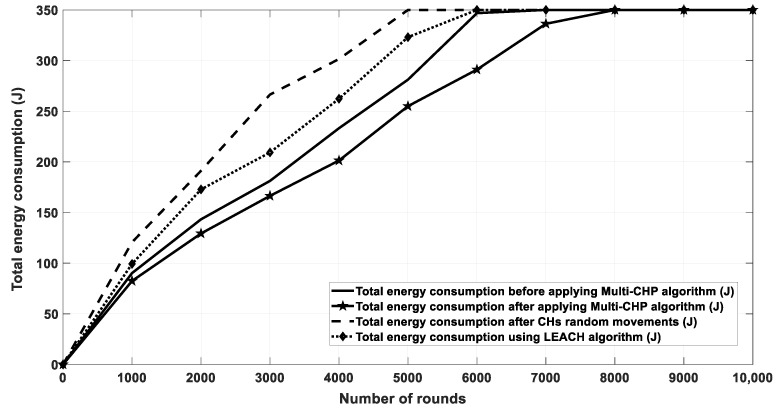
Energy consumption for the proposed algorithm and other related work.

**Figure 17:**
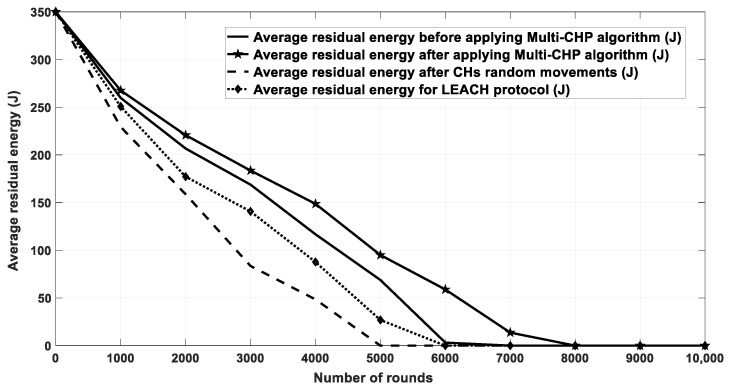
Residual energy for multi-CHP and other related work.

Figure 16 shows the energy consumption for network nodes over 10,000 rounds for four cases. The first case was for the current location of CHs without any movement. The second one was for random movement of CHs within 10% of their coverage area. The third one was for the proposed multi-CHP algorithm with CHs moved to their optimal locations with respect to the cluster SNs, and the fourth one for the LEACH protocol. As seen from the figure, the proposed multi-CHP algorithm had lower energy consumption over transmission rounds than the others. For example, the decrement percentage in energy consumption was equal to 11% and 5%, compared to the LEACH and static spiral CH allocation, respectively. Figure 17 displays the remaining energy after each iteration; similar to the previous results, the proposed algorithm had higher remaining energy than the other related work.

**Figure 18:**
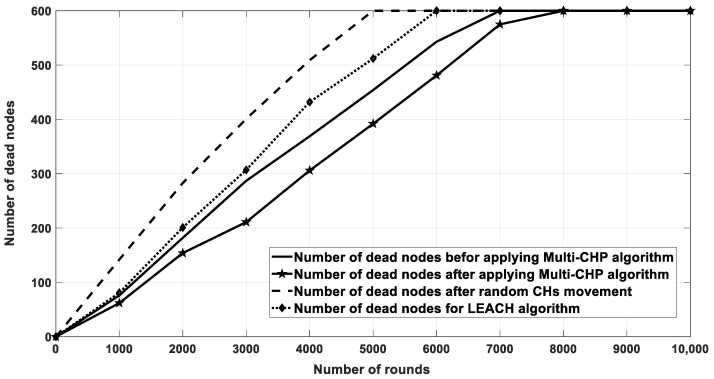
The number of dead nodes after each iteration.

The last comparison for this scenario shows the number of dead nodes after each round. As aforementioned, the node becomes dead if it consumes the majority of its transmission power, which directly effects the network lifetime. The network lifetime is calculated when all nodes in the network consume their energy. As demonstrated in Figure 18, the proposed algorithm has a longer lifetime than in the other scenarios and approaches. Further, Table 3 shows the FND, HND, and LND for different scenarios, which again showed that the proposed algorithm preserved the nodes energy for longer periods when compared with that of the other related work.

### 4.3. Multi-CHP vs. Dynamic Base Station Positioning Algorithm

In this section, the multi-CHP is compared with a Dynamic Base Station-Positioning algorithm proposed in [34], which assumes the BS a mobile node, and used the location and residual energy for CHs in the network to determine the best location for the BS. The process of determining the location for BS was repeated every transmission iteration. The simulation parameters used were the same as the ones in Table 1 with the following changes, which were made to ensure a fair comparison with the results of the Dynamic Base Station Positioning algorithm [25]. The total number of nodes was set to 100, and the AoI was set to 100 × 100 m^2^. The total initial energy of all nodes for multi-CHP and for Dynamic Base Station Positioning algorithm was equal to 25 J, which was distributed as follows:

- Each SN in Dynamic Base Station Positioning algorithm has an initial energy equals to 0.25 J.
- Based on Equation (15), the number of CHs in multi-CHP equals to 10, and the initial energy of each CH node is equal to 0.88 J.
- Each SN in multi-CHP has an initial energy equals to 0.18 J.

**Figure 19:**
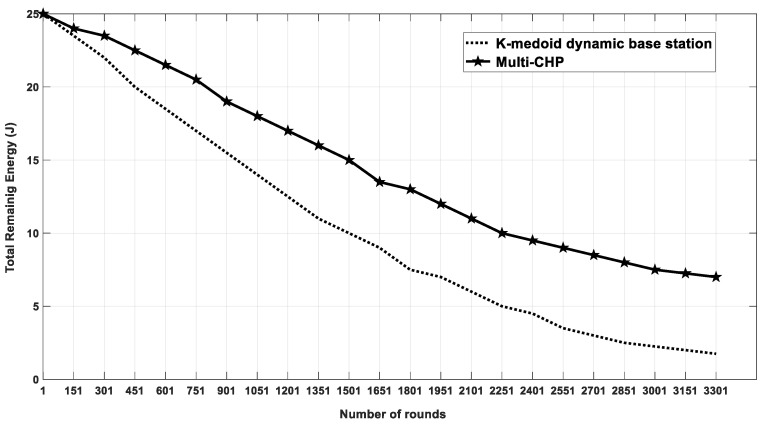
The average residual energy as a function of the simulation rounds for multi-CHP and k-medoid with dynamic base station positioning.

Finally, the cluster radius and the packet size are set to 25 m, 100 bytes, respectively [34]. Figure 19 shows the total residual energy for each multi-CHP and K-medoid with the dynamic base station-positioning algorithm over variable number of rounds. The figure shows an enhancement in the total residual energy of around 17% for multi-CHP when compared with that of the Dynamic Base Station Positioning algorithm.

### 4.4. Multi-CHP vs. SMOC

In this section, the performance of the multi-CHP is compared with one of the recent data gathering protocols called SMOC [25]. The simulation parameters used were the same as the ones listed in Table 1 with the following changes, which were made to ensure a fair comparison with the results of the SMOC algorithm [25]. The total number of nodes was set to 100, the AoI was set to 100 × 100 m^2^, and the SNs initial energy (E_no_) was equal to 0.5 J. Finally, the packet size was set to 2048 bits.

In SMOC, a single mobile sink moved in a predefined spiral path over the sensing field to collect data from sensor nodes. Initially, the cluster formation process starts when the nodes were deployed. All the clusters were formed in the order of the center; therefore, the sink could pass them in a spiral pattern. The sink started moving from the center of the network and traveled to the next point with predefined location points of the sensor field. The sink stopped by the first cluster and waited, in the meantime, the CH transmitted all the data to the sink and updated the location of the sink to the next CH. After receiving data from the first cluster, the sink moved to the next one. The sink could stay at any cluster for different amounts of time depending on the amount of data packets transmitted by each CH. This variation of time will be reflected positively on the data delivery ratio and network lifetime [25].

**Figure 20:**
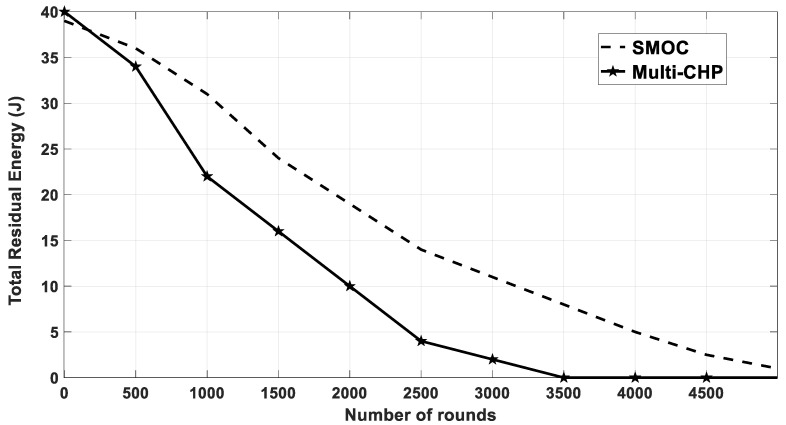
The average residual energy as a function of simulation rounds for multi-CHP and spiral mobility based on optimized clustering (SMOC).

Figure 20 shows the total residual energy for the multi-CHP and the SMOC over various number of rounds. The figure shows that the nodes in multi-CHP consumed their energy completely at round 3500, and at round 5000 in SMOC case.

The obtained results for multi-CHP lifetime in comparison with SMOC can be refuted as follow:

- The amount and degree of mobility required to move the mobile sink in SMOC was very high because of the continuous movement on the spiral trajectory over long distances, while multi-CHP had much lower mobility since the mobility was limited and within each cluster. Having a highly mobile sensor node that can traverse long distance has several challenges. It must have the ability of having efficient obstacle avoidance functionality, which may affect the reliability of the system if the mobile node was trapped with an obstacle and could not move to the next CH. Further, the amount of energy needed for such a mobile node is normally high when compared with a node with limited mobility. In addition, the delay in collecting the data from the CH will be higher since it depends on the speed of the mobile sink node. Finally, the complexity, cost, and hardware design and implementation of a high mobile sink node proposed in SMOC was very high when compared with a low mobile CH node proposed in the multi-CHP algorithm, which makes our proposed solution more practical from implementation point of view.
- The communication model in multi-CHP depended on the SNs sending their data to CHs and then the CHs sending it to the BS, and that allowed the CHs to pre-process the data (e.g., compress them) before sending them directly to the sink node; thus, reducing the transmission time. This model can be used not only for simple data gathering process, but also to enable the CHs to perform advanced data analysis, processing, and decision making before sending the data to the sink node, which results in a more optimized performance. On the other hand, SMOC is efficient in data gathering process only, and is not optimized for more advanced data analysis operation. For instance, suppose the sensors are monitoring an AoI to detect a critical event such as fire for example. In multi-CHP network mode, all the nodes within the cluster will send their sensed data to their perspective CHs, which can be designed to process the data, analyze it, and detect if there is a critical event or not. In case of detecting a critical event, the CH will send an alert message to the sink node, which can be sent directly or using other CHs in a multi-hop manner, until the alert message reaches the sink node. While in SMOC, all the nodes may send the data either to the CH or to the sink node directly, which will generate high unnecessary traffic at the sink side. Particularly, this traffic may, or may not, correspond to a critical event; thus, wasting the network resources and reducing its efficiency.
- The main aim of multi-CHP was to provide a methodology for CHs re-positioning and optimization, which can be used in any clustering algorithm to improve its performance as far as the CH is equipped with mobility functionality. On the other hand, the aim of SMOC was to provide a method for data gathering protocol. Hence, both algorithms have different main targets.

### 4.5. Multi-CHP vs. EECPEP-HWSN and DEEC

**Figure 21:**
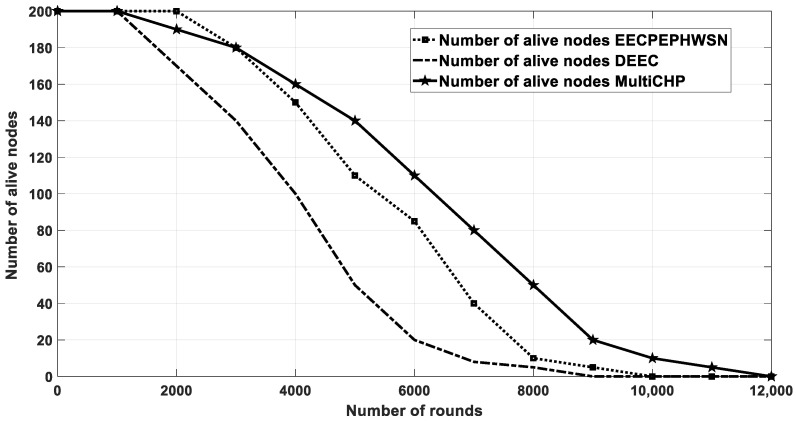
The number of alive nodes after each iteration.

In this section, the multi-CHP is compared with the DEEC [18], and the EECPEP-HWSN [19] protocols. In EECPEP-HWSN, the network consists of *N* randomly deployed sensor nodes on M × M m^2^ AoI. The simulation parameters used were the same as the ones in Table 1 with the following changes, which were made to ensure a fair comparison with the results of the EECPEP-HWSN and DEEC protocols. The total number of nodes was set to 200, the AoI was set to 200 × 200 m^2^, the SNs total initial energy was equal to 335 J. Finally, the packet size was set to 4000 bytes. As depicted in Figure 20 and Figure 21, the proposed multi-CHP outperformed the EECPEP-HWSN and DEEC protocols in terms of the number of alive nodes and network remaining energy, especially at large number of rounds, which verified the effectiveness of the proposed algorithm.

**Figure 22:**
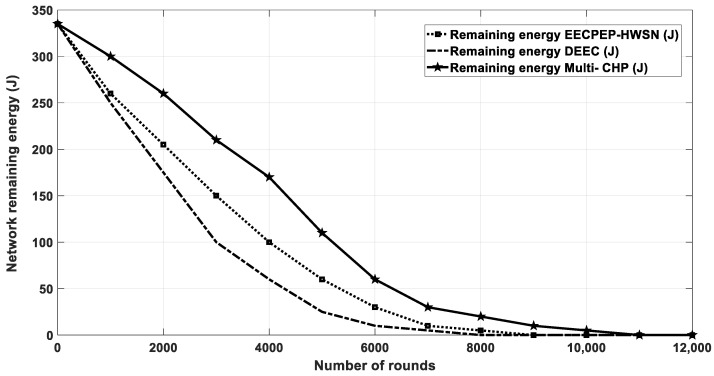
Residual energy after each iteration.

The network lifetime is calculated when all nodes in the network consume their energy. As demonstrated in Figure 21, the proposed algorithm had longer lifetime than the other scenarios and approaches. Further, Table 4 shows the FND, HND, and LND for different scenarios, which again showed that the proposed algorithm preserved the nodes energy for longer periods when compared with that of the other related work. Finally, Figure 22 depicts the nodes’ residual energy after each integration, which again shows a superior performance when compared with the other algorithms.

## 5. Conclusions and Future Work

Energy saving, SNs deployment, and communication interference mitigation are critical issues in implementing WSNs applications. The type, optimal number of nodes and clusters, and the locations of the sensor nodes have to be judiciously planned. This is to facilitate the enhancement of the performance requirements such as sensing, coverage, connectivity, network lifetime, and reliability of the data. This problem is particularly important for WSN applications, especially in large scale WSNs, where a large number of nodes will be employed. Due to the hard placement and locations adjustment of the network nodes by human or machines in some applications, mobile nodes are used to find the best locations to maximize the SNs lifetime.

In this paper, an algorithm to find the optimal location of the CH is proposed for single and multiple clusters, with the main objective of minimizing the overall energy consumption and extending the lifetime of the whole network. Theoretical analysis and extensive simulation results verified the correctness and high efficiency of the proposed algorithm when compared to the related work in literature. The main advantage of the proposed algorithm that it can be applied to any clustering algorithm used in mobile WSNs, where the nodes first find the CH according to the CH selection criterions, then the selected CH can change its location according to the proposed algorithm such that it minimizes the path-loss, which ultimately will reduce the overall energy consumption and improve the nodes’ life time. As for future work, we are running towards implementing the system in a real environment, which will enable us to evaluate the practicality of the system and propose corrective measures to the proposed algorithm, particularly about the mobility aspect, which is a challenging problem especially in rough terrain environments.

## Figures and Tables

**Table 1:** Simulation parameters used in CHP.

Description	Value
Number of nodes	(10–100) first scenario (40) second scenario
Area	100 × 100 m^2^
Transmitter electronics (ETX-elec) Receiver electronics (ERX-elec) (ETX-elec) = (ERX-elec) = (Eelec)	50 nJ/bit
Energy consumed by the amplifier to transmit at a shorter distance $\epsilon_{fs}$	10 pJ/bit/m^2^
Energy consumed by amplifier to transmit at a longer distance $\varepsilon_{amp}$	0.0013 pJ/bit/m^4^
Initial energy	SNs $E_{\left(non-CH\right)}$ = 0.5 J CH nodes *E_CH_* = 2.5 J
Sink node location	(0,0)
Packet size	2000 bits

**Table 2:** The FND, HND, and LND metrics for the different scenarios of Figure 13.

Scenario/Metric	FND	HND	LND
Before applying CHP	70	800	1400
After applying CHP movement	100	1150	1800
After CH random movement	50	650	1400
LEACH	80	950	1600

**Table 3:** The FND, HND, and LND metrics for the different scenarios of Figure 18.

Scenario/Metric	FND	HND	LND
Before applying multi-CHP	60	3200	7000
After applying multi-CHP movement	80	4000	8000
After CH random movement	20	2200	5000
LEACH	55	3200	6000

**Table 4:** The FND, HND, and LND metrics for the different scenarios of Figure 21.

Scenario/Metric	FND	HND	LND
Multi-CHP	1180	6380	11,820
EECPEP-HWSN	1931	5200	9284
DEEC	1136	4000	8375
